# Molecular Aspects of Mycotoxins—A Serious Problem for Human Health

**DOI:** 10.3390/ijms21218187

**Published:** 2020-10-31

**Authors:** Edyta Janik, Marcin Niemcewicz, Michal Ceremuga, Maksymilian Stela, Joanna Saluk-Bijak, Adrian Siadkowski, Michal Bijak

**Affiliations:** 1 Biohazard Prevention Centre, Faculty of Biology and Environmental Protection, University of Lodz, Pomorska 141/143, 90-236 Lodz, Poland; edyta.janik@unilodz.eu (E.J.); marcin.niemcewicz@biol.uni.lodz.pl (M.N.); 2 Military Institute of Armament Technology, Prymasa Stefana Wyszyńskiego 7, 05-220 Zielonka, Poland; ceremugam@witu.mil.pl; 3 CBRN Reconnaissance and Decontamination Department, Military Institute of Chemistry and Radiometry, Antoniego Chrusciela “Montera” 105, 00-910 Warsaw, Poland; m.stela@wichir.waw.pl; 4 Department of General Biochemistry, Faculty of Biology and Environmental Protection, University of Lodz, Pomorska 141/143, 90-236 Lodz, Poland; joanna.saluk@biol.uni.lodz.pl; 5 Department of Security and Crisis Menagement, Faculty of Applied Sciences, University of Dabrowa Gornicza, Zygmunta Cieplaka 1c, 41-300 Dabrowa Gornicza, Poland; ASiadkowski@wsb.edu.pl

**Keywords:** mycotoxins, human health, contamination, molecular aspects

## Abstract

Mycotoxins are toxic fungal secondary metabolities formed by a variety of fungi (moulds) species. Hundreds of potentially toxic mycotoxins have been already identified and are considered a serious problem in agriculture, animal husbandry, and public health. A large number of food-related products and beverages are yearly contaminated by mycotoxins, resulting in economic welfare losses. Mycotoxin indoor environment contamination is a global problem especially in less technologically developed countries. There is an ongoing effort in prevention of mould growth in the field and decontamination of contaminated food and feed in order to protect human and animal health. It should be emphasized that the mycotoxins production by fungi (moulds) species is unavoidable and that they are more toxic than pesticides. Human and animals are exposed to mycotoxin via food, inhalation, or contact which can result in many building-related illnesses including kidney and neurological diseases and cancer. In this review, we described in detail the molecular aspects of main representatives of mycotoxins, which are serious problems for global health, such as aflatoxins, ochratoxin A, T-2 toxin, deoxynivalenol, patulin, and zearalenone.

## 1. Introduction

Mycotoxins are secondary metabolites synthesized by a variety of fungi (moulds) species such as *Fusarium*, *Aspergillus*, *Penicillium*, *Alternaria*, and *Claviceps*. They constitute a structurally diverse group of toxic and low molecular weight compound, which is generally less than 1000 Da [1,2,3]. Approximately 400 potentially toxic mycotoxins produced by more than 100 fungi species have been identified and it is considered that the most toxigenic to agriculture, animal husbandry, and public health are trichothecenes, ochratoxins, aflatoxins, zearalenone, fumonisins, patulin, and citrinin. Diseases caused by mycotoxins are called mycotoxicoses. Mycotoxins can enter human and animal bodies via food and feed. They contaminate many agricultural commodities. According to the Food and Agriculture Organization of the United Nations (FAO) report, 25% of global agricultural products are contaminated by mycotoxins each year, resulting in economic losses [4,5,6]. Growth of fungi and toxin production can occur at any stage of cultivation, harvesting, or storage, which leads to their contamination. Mycotoxin contamination is a global problem and is more common in countries where harvesting, production technologies, or storage conditions are inefficient. Additionally, exposure to moulds and mycotoxins may occur in buildings with high humidity. Mycotoxigenic mould growth is essential for mycotoxin production, but the presence of mould species does not indicate toxin production. Specific conditions are required for mycotoxins secretion. Temperature, water activity, pH, oxygen, and substrate composition affect the production of mycotoxin [7,8,9]. The optimal temperature for moulds’ growth varies from 20 °C to 37 °C and for the production of toxin it is 25.5 ± 5.5 °C. Mycotoxin secretion can occur at lower temperatures (below 10 °C); however, the time of production is longer and toxin concentration is reduced. Apart from temperature, a significant factor for mycotoxin production is water activity, and the optimal range of this parameter varies from 0.83 to above 0.9 a_w_ [8,9,10]. Additional factors increasing moulds’ growth and mycotoxin production are high relative humidity (70–90%) and moisture content (20–25%). Soil and air are natural environments for some mould species, which makes protection against mycotoxin contamination problematic. However, controlling factors Tat (positively charged Arginine-rich peptide) leads to mould growth and toxin secretion which may be effective in contamination reduction [11]. Mycotoxins are generally found in cereal (wheat, corn, feed), spices and seeds. However, when animals are fed, mycotoxin-contaminated feed can accumulate in their tissues, which indicates that animal-derived food like eggs, milk, or meat can also become contaminated [12,13]. Mycotoxin contamination is considered an unpredictable and inevitable problem due to its resistance to high temperatures and chemical or physical treatments. For this reason, standard cooking is not sufficient for mycotoxin elimination [14]. Cereals have always been a main source of mycotoxins in human and domestic animals’ diet. Ergot alkaloid intoxication, called ergotism, was very common in Central Europe in the Middle Ages. From the 9th to 14th century, the outbreak of ergotism was widespread in the eastern regions of France, Germany, and Russia. The disease appeared in two characteristic forms: convulsive ergotism, manifested in trembling muscles, convulsions, hallucinations, and gangrenous ergotism, manifested by severe vasoconstriction leading to auto-amputation of the limbs. These two types of disease could occur concurrently. In Japan, in the second half of 19th century and in the 20th century, acute cardiac beriberi was described. A disease characterized by palpitations, heart distress, progressive paralysis, and respiratory failure symptoms was caused by *Penicillium* species of toxins found in rice. Moreover, other *Penicillium* species observed in corn were the cause of animal toxicosis in Nebraska in the USA in the early 20th century. Moreover, the alimentary toxic aleukia (ATA) outbreak appeared in the Soviet Union in the 1940s. This mycotoxicosis was caused by the *Fusarium* species (*F*. *poae*, *F*. *sporotrichioides*), mainly produced by T-2 mycotoxin. It was initially thought that clinical manifestations were more likely a result of radiation rather than food poisoning. Furthermore, aflatoxins were responsible for the well-known ”X” turkey disease in England in the 1960s [15,16]. It should be emphasized that some of the toxic fungal species can remain active for thousands of years. In 1962, poisonous fungi was found in Egyptian ancient tombs and mummies. It is considered that fungi were responsible for the deaths of people involved in the discovery of the Tutankhamen tomb [17]. Today, mycotoxins also have a definitely negative impact on human or animal health and they can even cause death. However, their most common effects on health are nephrotoxicity, hepatotoxicity, immunosuppression, carcinogenicity, and teratogenicity. Table 1 lists the most important toxins and toxic effects of poisoning [18,19]. The economic losses associated with mycotoxins contaminations are significant. Losses resulting in Fusarium head blight outbreak and deoxynivalenol contamination in 1996 in Ontario were estimated at approximately 145 million US dollars [15].

The mycotoxins are also very attractive to terrorists for use in acts of bioterrorism. Most of them can be obtained very easily from environmental sources, and since culturing systems and extraction equipment are cheap and easily available, they can even be constructed at home [20]. Many reviews focus on information on the presence of mycotoxins in various agricultural products, mycotoxicity risk to human and livestock health, mycotoxicosis mitigation strategies, prevention, and control to ensure consumer safety. In our review, in addition to general information on fungal toxins, we want to present the problem of mycotoxins in molecular terms, which not only may provide the basis for a better understanding of the activity and action of those toxins, but also help in the development of detailed protocols related to a quick response to mycotoxins poisoning.

## 2. Aflatoxins

**Figure 1:**
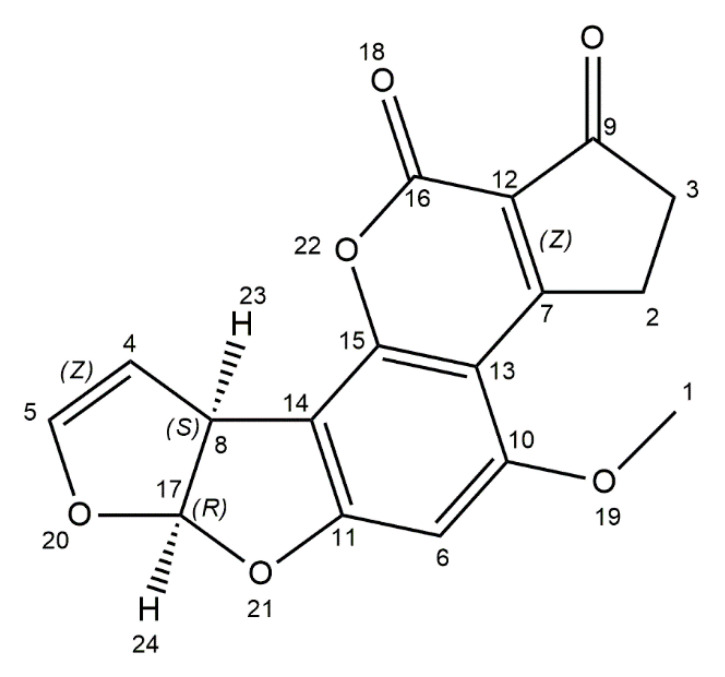
Chemical structure of aflatoxin B1 (structure generated from InChI code available on https://pubchem.ncbi.nlm.nih.gov/) (accessed on 16 August 2020).

Aflatoxins are produced by *Aspergillus* species such as *A*. *flavus*, *A*. *parasiticus*, and rarely *A*. *nomius*. Among 18 different types of aflatoxins, there are four commonly occurring: B_1_ (AFB_1_), B_2_ (AFB_2_), G_1_ (AFG_1_), and G_2_ (AFG_2_) [21,22,56]. The AFB_1_ is defined as the most common contaminant of foods and the most carcinogen and mutagen potent. According to the International Agency for Research on Cancer (IARC), AFB_1_ is classified as group 1 carcinogen (carcinogenic to humans). Human exposure to aflatoxin B_1_ is especially dangerous in populations with a high rate of hepatitis B virus (HBV), because it is estimated that the risk of liver cancer from AFB_1_ exposure in HBV-positive people is 30 times higher than in the HBV-negative [35]. It is considered that AFB_1_ causes up to 28% of worldwide cases of hepatocellular carcinoma (HCC), which is the most frequent form of liver cancer [57]. Chemically, aflatoxins are coumarin derivates, containing a fused dihydrofurofuran moiety. The structure of AFB_1_ is distinguished by the cyclopentenone ring fusion to the coumarin lactone ring (Figure 1). Moreover, AFB_1_ is freely soluble in polar organic solvents, slightly soluble in water, and insoluble in nonpolar solvents. This toxin is also characterized by instability to pH conditions, such as <3 or >10, and UV light [58,59]. Aflatoxicosis is a result of consumption of contaminated food, which can cause immunosuppression, stunting in children and cancer. Tropical and subtropical countries are more exposed to aflatoxicosis, because the level of food contamination with mycotoxins is not sufficiently monitored. Aflatoxins contribute to the contamination of maize, peanuts, wheat, barley, oilseeds, and spices, but also milk, dairy products, meat, and eggs as a consequence of mouldy feed consumed by livestock [21,23,60,61]. *Aspergillus* contamination can occur at pre- and post-harvest stages. Fungal growth can occur also in unsuitable storage conditions [60,62]. Optimum conditions for *Aspergillus* growth in peanut kernels and on polished rice are 28–40 °C and a_w_ 0.94–0.99, and for aflatoxins production they are 25–33 °C and a_w_ 0.92–0.96. The maximum amounts of aflatoxin B_1_ in peanut kernels is at 28 °C, a_w_ 0.96, and on polished rice optimal values are 33 °C and a_w_ 0.96 [63]. AFB_1_ is absorbed in the small intestine, transferred to blood stream, and then transported by plasma proteins and red blood cells to the liver. In liver cells, toxin is metabolized by microsomal-mixed function oxidase (MFO) enzymes, which belong to the Cytochrome P450 (CYP450) superfamily. AFB_1_ is converted to a more toxic, highly reactive 8,9-exo-epoxide and 8,9-endo-epoxide metabolites. The 8,9-exo-epoxide has a high affinity to bind to the DNA and then form the 8,9-dihydro-8-(*N*’-guanyl)-9-hydroxy-AFB1 (AFB1-*N*-Gua) adduct, the formation of which leads to DNA mutations. The 8,9-exo-epoxide also binds to other macromolecules like RNA and proteins, which leads to inhibition of RNA, DNA, protein syntheses, and cellular function disorders. This epoxide can be involved in enzymatic and non-enzymatic conversion in AFB1-8,9-dixydroxydiol that can bind protein such as albumin, or can be converted in the aflatoxin dialdehyde and excreted via urine as a result of aflatoxin aldehyde reductase action. In the endoplasmic reticulum (ER) of liver cells, AFB_1_ is also hydroxylated to fewer toxic metabolites: aflatoxin M_1_ (AFM_1_), aflatoxin Q_1_ (AFQ_1_), and aflatoxin P_1_ (AFP_1_) [59,64,65,66]. In ruminants fed with contaminated feed, a part of the AFB_1_ is degraded by ruminal fluid microbiota and then transformed into an 18-times less toxic metabolite called aflatoxicol. About 1–2% of the toxin is absorbed in the gastrointestinal tract, next, by passive diffusion, it is hydroxylated in the liver and then it forms AFM_1_ metabolite, which occurs in blood and is finally secreted in milk. Ruminants, as a consequence of their characteristic four-chamber stomachs, are considered to be less susceptible to mycotoxins than monogastric species, because mycotoxins can be partially eliminated by the rumen microbiota. However, the ruminal microbial detoxification varies depending on dietary changes or as a result of metabolic diseases [64,67,68]. The maximum limit of AFB_1_ has been set by the European Union at 2 μg/kg in all cereals and all cereal-derived products. China also has regulated the maximum limit of aflatoxin B_1_ at 5 μg/kg in barley, wheat, and their products, 20 μg/kg in corn and corn products [24]. The Food and Drug Administration (FDA) has set a maximum level for aflatoxins in dairy animal feed at 20 μg/kg [25].

## 3. Ochratoxin A

**Figure 2:**
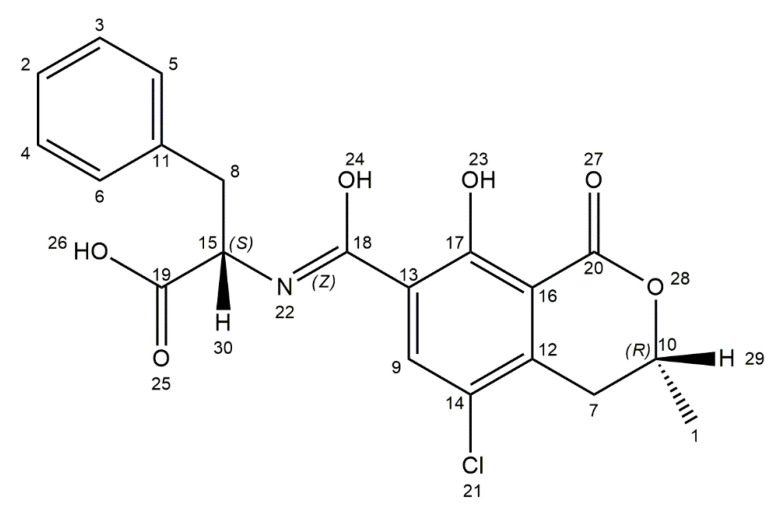
Chemical structure of ochratoxin A (structure generated from InChI code available on https://pubchem.ncbi.nlm.nih.gov/) (accessed on 16 August 2020).

Ochratoxin A (OTA) is produced by several fungal species including *Aspergillus ochraceus*, *A*. *carbonarius*, *A*. *niger*, *Penicillium verrucosum*, *P*. *nordicum*, and *P*. *viridicatum* [26]. The optimal conditions for growth of OTA-producing fungi range from 15 to 40 °C and 0.77 to 0.99 water activity. The optimum temperature for toxin production for *A*. *niger* on maize kernels is 15–40 °C and for *A*. *carbonarius*, it is 15–35 °C [69,70,71]. The conditions under which *A*. *ochraceus* can produce ochratoxin A in coffee beans are a_w_ 0.97–0.99 and 25–30 °C [69]. OTA is more common and the most toxic among ochratoxins, like ochratoxin B (OTB) or ochratoxin C (OTC) [72]. Different types of food are sources of exposure to OTA, including cereals (oats, maize, wheat, barley), cereal products, coffee beans, dried fruits, beer, grape juice, wine, as well as nuts, cacao products, and spices. *A*. *carbonarius* is the main OTA producer in wine and wine-dried fruits [27,28]. In addition, this toxin is found in milk products, eggs, and meat [73,74]. The chemical name of OTA is L-phenylalanine-*N*-[(5-chloro-3,4-dihydro-8-hydroxy-3-methyl-1-oxo-1*H*-2-benzopyrane-7-yl)carbonyl]-(*R*)-isocoumarin. It is a pentaketide derived from the dihyrdocoumarins family, linked by a peptide bond to β-phenylalanine (Figure 2). It is a weak organic acid, which in neutral and acid conditions are soluble in polar organic solvents (chloroform, alcohols). In alkaline pH, it is soluble in aqueous sodium bicarbonate solution. The crystalline structure of OTA varies from white to colorless, and this toxin exhibits blue fluorescence in alkaline conditions and green fluorescence in acidic environment [75,76,77].

Many studies have shown negative effects of OTA on human and animal health. It induces several toxic effects like nephrotoxicity, hepatotoxicity, genotoxicity, teratogenicity, immunotoxicity, and neurotoxicity. The toxin has a half-life of 840 h in blood after oral ingestion. OTA is absorbed from the small intestine, enters the circulation, and binds to the serum albumin in plasma. In the human circulatory system, 99.8% of ochratoxin A is present in the albumin-bond form. Erythrocytes contain insignificant amounts of OTA. Then, it is distributed to kidneys, liver, muscle, brain, and fat [29,30,78,79]. Kidneys are the primary target [80] in which using transmission electron microscopy focal tubular cell proliferation, multiple adenoma-like structures in layers of the renal papilla and in convoluted tubules were observed [81]. However, the presence of OTA has also been found in bone marrow, skin, adrenal medulla, and cortex or myocardium [82]. Different mechanisms of OTA action have been observed. The toxin causes G2/M phase cell cycle arrest, apoptosis, necrosis, inhibition of microtubule assembly, cell division processes, or protein syntheses. Several studies have shown that OTA induces reactive oxygen species (ROS) generation leading to oxidative stress and ROS-mediated apoptosis, as well as DNA adducts and DNA single-strand breaks [79,83,84]. Shin et al. have demonstrated that OTA causes a decrease in glutathione (GSH), which is an important antioxidant. OTA treatment causes also ROS, lipid peroxides, and nitric oxide (NO) generation [85]. Another study has shown that exposure of chickens to OTA decreased their number of lymphocytes, leukocytes, and throbocytes, modified the mucosal architecture of duodenum, jejunum, and also ileoceacal junction, caused a reduction in the intestinal TCR1, TCR2, CD4+, CD8+ lymphocyte population, and led to enterocyte apoptosis [86]. The neurotoxic properties of OTA are the result of inhibition of human astrocyte cell line proliferation and differentiation. An increase in mitochondrial and cytosolic calcium concentration also appears as a consequence of calcium overload—cells’ death occurs. OTA inhibits expression of glutamate transporter 1 (GLT1), glutamate aspartate transporter (GLAST), that leads to reducing glutamate absorption by astrocytes, which play a potential role in stability or induction of neurodegenerative diseases like Parkinson’s and Alzheimer’s, as well as neuron motor degenerations [87,88]. Bhat et al. have shown that in neuronal (Neuro-2a) cell line, OTA causes ROS generation resulting in c-jun amino-terminal-kinase (JNK)-mediated caspase-dependent apoptosis [89]. The IARC has classified ochratoxin as a group 2B carcinogen (possible human carcinogen) [90]. Various institutes and committees have developed limits on the OTA content in different types of food. The European Union has set a maximum limit of ochratoxin A at 3 μg/kg for cereal products, 5 μg/kg for unprocessed cereals, 10 μg/kg for dried fruits, and 15 μg/kg for spices [31,32]. The European Commission established a maximum limit of OTA at 5 ng/kg in coffee beans, 10 ng/kg in instant coffee, 0.5 μg/kg in cereal-based food for infants and children, and 2 μg/kg in wines [33,34]. Furthermore, the Joint FAO/WHO Expert Committee on Food Additives (JECFA) has set the provisional maximum endurable consumption of OTA at 0.1 μg/kg b. w. per week [35].

## 4. T-2 Toxin

**Figure 3:**
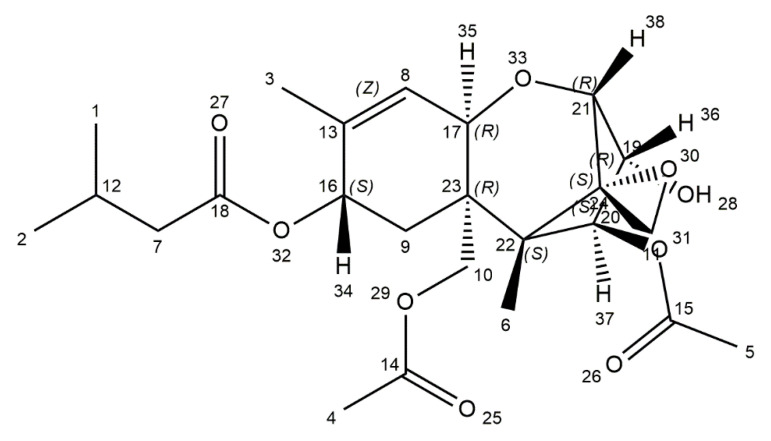
Chemical structure of toxin T-2 (structure generated from InChI code available on https://pubchem.ncbi.nlm.nih.gov/) (accessed on 16 August 2020).

T-2 toxin is one of the most toxic mycotoxins, mainly produced by *Fusarium sporotrichoides*, *F*. *poae*, *F*. *acuminatum*, and *F*. *equiseti,* which are mainly found in regions with cold climate and wet storage conditions [36,91]. The World Health Organization categorized T-2 toxin as an unavoidable contaminant in agricultural products, human food, and animal feed as early as in 1973. T-2 toxin naturally occurs in cereals, especially in wheat, oats, barley, and also in cereal-based products. It makes this toxin harmful to human and animal health [37]. Contamination of overwintered wheat caused an outbreak of alimentary toxic aleukia (ATA) in the 1930s in the former Soviet Union and was related with other gastrointestinal problems [92]. The etiology of Kashin–Beck disease (KBD) is still unclear, but it can be suspected that T-2 mycotoxin is the cause of this disease. In Chinese villages, which are endemic for KBD, the presence of T-2 toxin is relatively high with an average range of 78.91 μg/kg in wheat and 47.47 μg/kg in flour [93,94,95]. Pleadin et al. have presented that in unprocessed cereals and cereal-based products coming from Croatia and Bosnia and Herzegovina, the proportion of contamination with T-2 ranged from 26.9% to 81.6% [96]. T-2 mycotoxin molecular weight is 466.51 and it occurs as white, needle-like crystals. It has a distinctive tetracyclic sesquiterpenoid 12,13-epoxytrichothec-9-ene ring in common, and a 12,13-epoxy ring, which has a crucial function for the toxicity. The chemical structure is characterized by a hydroxyl (OH) group at the C-3 position, acetyloxy (-OCOCH3) groups at C-4 and C-15 positions, an atom of hydrogen at C-7 position, and an ester-linked isovaleryl [OCOCH2CH(CH3)2] group at the C-8 position (Figure 3) [37,97].

The metabolism of T-2 mycotoxin has been studied in various in vitro and in vivo experiments. The main biotransformation pathway of T-2 toxin is deacetylation of the C-4 acetyl group, leading to a conversion to HT-2 toxin. HT-2 toxin has been detected in isolated kidney microsomes, liver, and spleen of various animal models as the sole metabolite of T-2 toxin. Other reactions usually connected with metabolism of this toxin in mammals are oxidation (e.g., 3′-hydroxy-HT2 and 3′-hydroxy-T2), hydrolysis (e.g., neosolaniol, T2-triol, and T2-tetraol), de-epoxidation (e.g., de-epoxy-3′-hydroxy-HT2, de-epoxy-T2-triol, and de-epoxy-HT2), and glucuronide conjugation of biotransformation products such as HT-2 and neosolaniol. De-epoxidation is an essential detoxification mechanism and, with metabolic changes (e.g., conjugation) of the hydroxyl group at C-3, it has an effect on reducing the toxicity of trichothecenes. In vitro studies with African green monkey kidney cell line (VERO) and Chinese hamster ovary cell line (CHO) identified traces of T-2 triol, T-2 tetraol, and several other unknown metabolites. Studies with human blood cells have shown the metabolism of T-2 toxin to HT-2 toxin and neosolaniol as second metabolite by carboxylesterases activity. The amount of both metabolites was equal. In contrast, experiments with human liver homogenates have demonstrated HT-2 toxin as the only metabolite [98,99]. The lipophilic nature of this toxin implies that it is easily absorbed through the gut, skin, and pulmonary mucosa. T-2 toxin impacts the vascular system, leading to dilation and swelling of micro vessels, as well as damage of the plasma membrane and the blood vessel wall. Central nervous system disorders caused by T-2 toxin poisoning cause lethargy, ataxia, and emesis in humans and animals. Various reports suggest that exposure to T-2 toxin can change the concentration of neurotransmitters in the brain. Dermal exposure to this toxin in mice increases the blood–brain barrier (BBB) permeability and it is related to the activation of matrix metallopeptidase 9 (MMP-9) and proinflammatory cytokines IL-1 α, IL-1β, and TNFα in periphery and in the brain [39]. Chaudhary et al. have showed that T-2 mycotoxin exposure via percutaneous and subcutaneous route causes notable oxidative brain damage as a consequence of increased lipid peroxidation, depletion of hepatic glutathione, changes in antioxidant enzymes activity, and protein oxidation [100]. Kang et al. have demonstrated that mice exposure to T-2 toxin during pregnancy and lactation can lead to an increase in the lipid content in young mice’s liver tissues and serum. Furthermore, disruptions in bile acid metabolism may lead to lipid accumulation in the liver and as a consequence result in young mice’s liver disfunction [36]. The significant toxicological effects of T-2 mycotoxin is dermal irritation and wound forming. Skin exposure to this toxin induces a spectrum of damages from erythema to necrosis. Skin inflammation, fibroblast cell destruction in skin, and skin damages are similar to the detrimental effects of radiation. Inhibition of the DNA, RNA, and protein synthesis are considered to be the main cause of dermal damages [38]. T-2 toxin has a toxic effect on the immune system. It decreases the production of IL-2 and the expression of plasma IFN-γ and can upregulate the mRNA expression of IL-1β, IL-6, and TNF-α, depending on the dose in RAW264.7 cell line, which is a model of mice macrophage cells [93]. The European Commission has established a tolerable daily intake of of 100 ng/kg body weight for T-2 toxin [40].

## 5. Deoxynivalenol

**Figure 4:**
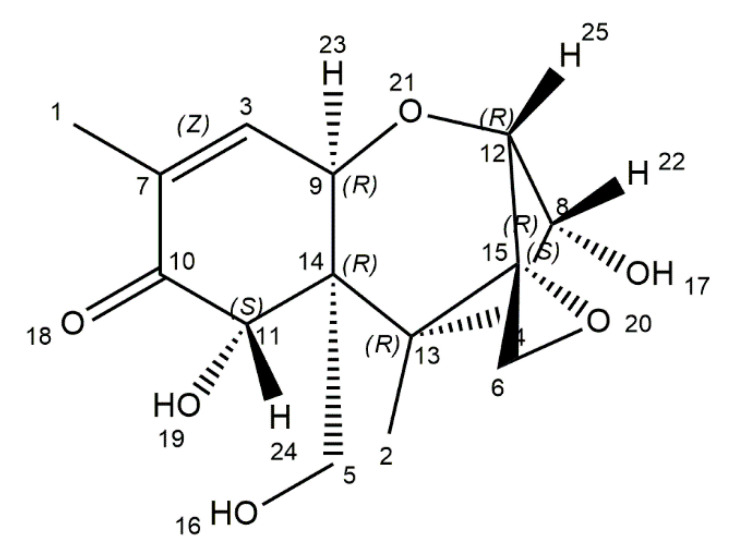
Chemical structure of deoxynivalenol (structure generated from InChI code available on https://pubchem.ncbi.nlm.nih.gov/) (accessed on 16 August 2020).

Deoxynivalenol (DON), also known as vomitoxin, is mainly produced by *Fusarium graminearum* and *F*. *culmorum* [41,42]. These fungi are essential plant pathogens, which grow on field crops and cause a disease called *Fusarium* head blight (FHB). DON can contaminate various types of food or feed and unprocessed grains, especially in temperate regions. DON is one of the most frequently occurring mycotoxins in European food and feed. In addition, 25% of global crops production is considered to be contaminated with this toxin. The highest level of this toxin is observed in maize, wheat, and derived products [43,45,101]. The chemical name of DON is 12,3-epoxy-3α,7α,15-trihydroxytrichothec-9-en-8-on (Figure 4). The molecular structure contains 3 free hydroxy groups (-OH), which are associated with its toxicity. It resembles colorless, fine needles, soluble in water and polar organic solvents (ethanol, methanol, chloroform, acetonitrile) [102]. DON remains stable in high temperatures, and at 150–170 °C, the toxin is not eliminated [43].

DON is called vomitoxin. The strong emetic/anorectic effects of DON are associated with two major mediators: proinflammatory cytokines and secretion of satiety hormones, which can activate receptors in the abdominal vagal afferent. The emetic effect was first observed in contaminated barley in 1972 in Japan [44,102]. Biological toxicity of DON was described in various in vitro and in vivo studies. Vomitoxin induces physiological irregularities, including digestive disorders as well as reproductive and endocrine disruptions [103]. The mechanism of toxicity of DON involves the inhibition of protein synthesis. The toxin interacts with peptidyl transferase enzyme, binding the 60S ribosomal subunit and then causes translation inhibition as well as ribotoxic stress. Another mechanism of toxicity is associated with the activation of a number of mitogen-activated protein kinases (MAPK), which are responsible for many effects of the toxin, including oxidative stress, apoptosis, inflammatory response, and endocrine disorders [104]. It has been shown that DON-induced toxicity in intestinal epithelial cells is based on MAPK activation in tandem with a decreased expression of the tight junction proteins (TJP) and the loss of barrier function [105]. Behrens et al.’s in vitro studies have shown that DON causes cytotoxic effect at the blood–brain barrier. A putative mechanism of action is based on increasing the cellular inflammation markers like MAPK and reducing the expression of claudins, which are significant to maintain the performance of TJP. It leads to reduced barrier integrity and, consequtively, increased permeability [101]. A different study has demonstrated that DON causes DNA damage as a result of direct action of toxin and by mechanisms like formation of DNA adducts from free radicals. DON causes lipid peroxidation and malondialdehyde (MDA) formation. MDA reacts with deoxy guanosine and deoxyadenosine in DNA, later forming DNA adducts, mainly the mutagenic M1G (pyrimidol(1,2-a]purin-10(3H)-one) [106]. Additionally, it has been shown that DON exposition can lead to toxin accumulation in various products of animal origin such as eggs, milk, fat, and muscle [45,107]. To reduce the risk of DON-induced effects, the JECFA has established a maximum daily limit of toxin intake at 1 μg/kg body weight [46]. DON has been classified by IARC in group 3 as it did not show carcinogenic effects in humans [108].

## 6. Patulin

**Figure 5:**
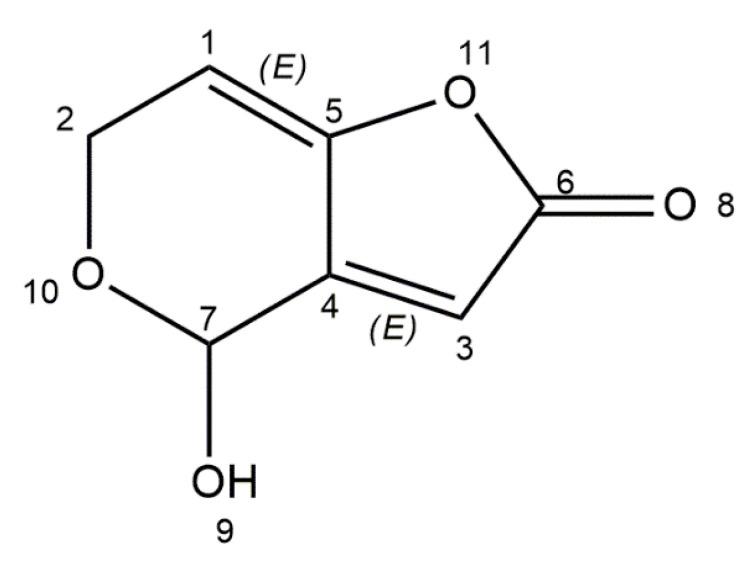
Chemical structure of patulin (structure generated from InChI code available on https://pubchem.ncbi.nlm.nih.gov/) (accessed on 16 August 2020).

Patulin is produced by fungi of the genera *Aspergillus*, *Penicillium*, *Byssochlamys*, and *Paecilomyces*. Thirteen species of *Penicillium* synthesize patulin among which are *P*. *expansum*, *P*. *carneum*, *P*. *coprobium*, *P*. *clavigerum*, *P*. *dipodomyicola*, *P*. *glandicola*, *P*. *concentricum*, *P*. *gladioli*, and *P*. *griseofulvum*. *Aspergillus* species like *A*. *clavatus*, *A*. *longivesica*, and *A*. *giganteus* are also patulin producers. Amongst *Byssochlamys* and *Paecilomyces* species, only *B. nivea* and *P. saturatus* can produce this mycotoxin [47,51]. *Aspergillus* species produce patulin in warm and humid environments (tropical and subtropical areas), while *Penicillium* species are responsible for toxin secretion in lower temperatures. *P*. *expansum* is a potential toxin producer in an apple’s pre- and post-harvest stage, whilst *Byssochlamys nivea* is related to patulin contamination in pasteurized fruit juices [109]. Patulin is a natural contaminant of fruits and vegetables, including apples, apple-derived products, plums, grapes, pears, pineapples, peaches, and tomatoes [48]. Predominantly, patulin contamination is associated with blue and soft rot, mainly caused by *P*. *expansum*. Humans are exposed to this toxin through consumption of contaminated food and beverages [110]. Many countries have done research and investigated into contamination related to patulin in apple and apple products. Studies have shown that in Belgium organic apple juice reveals higher levels of toxin than conventional juices. In Portugal, 23% of apple-derived products and 69% of rotten apples are contaminated with patulin. In India, investigations have shown that patulin is noticeable in 24% of apple juice samples, and in 16% of samples, more than 100 μg/L of toxin content was present, while the maximum level of patulin in apple juice is set by the WHO at 50 μg/L [111,112]. Patulin (4-hydroxy-4*H*-furo[3,2-c]pyran-2(6*H*)-one) is a polyketide lactone with low molecular weight (Figure 5). It is a white powder soluble in water and polar organic solvents (ethanol, methanol, acetone). It is also stable at high temperatures. Chemical studies have reported that the toxin is not eliminated from apple juice exposed to 80 °C for 20 min [113,114].

Initially, patulin was described as an antibiotic, but its toxic effects on animals were later observed. Exposure to patulin results in various acute and chronic health effects, including agitation, pulmonary congestion, hyperemia, dyspnea, edema, ulceration, and intestinal inflammation [115,116]. Patulin is highly toxic to the liver, gastrointestinal tract, kidneys, nervous system, and immune system [49]. The toxicity of patulin induces cell damage and cellular processes disruption through electrophilic reactivity, resulting in formation of adducts with nucleophiles like amino (-NH_2_) and sulfhydryl (-SH) groups. The adducts created with GSH, lysine-, cysteine-, histidine-, or α-amino-acid containing proteins are covalently cross-linked compounds [117,118]. Studies on kidney and intestinal cell lines have shown that patulin increases the level of intracellular ROS and also induces mitochondrial anion superoxide generation [119]. Patulin is also able to inhibit the activity of different enzymes, like aminoacyl-tRNA synthetases and RNA-polymerases [120]. Song et al. have demonstrated hepato- and genotoxic properties of patulin. The study has shown that mycotoxin causes serum alanine transaminase (ALT) and aspartate transaminase (AST) activity, induces lipid peroxidation, ROS generation, and decreases the GSH level in mice. Moreover, in bone marrow, patulin causes micronucleus and chromosomal aberration formation [50]. Other in vitro studies have shown nephrotoxic properties of patulin by activating p38 and JUN kinase in the HEK293 cell line [120]. Immunotoxicity of patulin has been confirmed in different studies. In mice, patulin increases a number of monocytes, NK cells and cytotoxic T cells, and decreases a number of lymphocytes and peripheral blood leukocytes [121]. According to IARC, patulin is a group 3 carcinogen as it is not carcinogenic to humans [122]. International agencies and institutions have introduced limits for patulin in various food products. WHO estimated the maximum limit of patulin at 50 μg/kg in apples, 50 μg/L in apple juice, and 10 μg/L in young children and infants’ apple-based food [51].

## 7. Zearalenone

**Figure 6:**
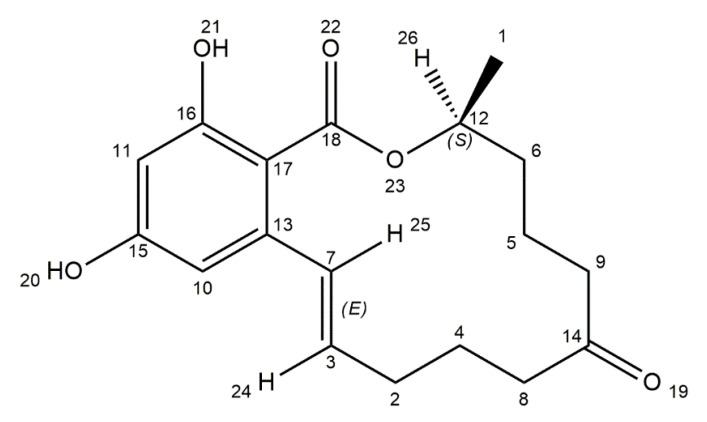
Chemical structure of zearalenone (structure generated from InChI code available on https://pubchem.ncbi.nlm.nih.gov/) (accessed on 16 August 2020).

Zearalenone (ZEA) is a nonsteroidal estrogenic mycotoxin, produced by *Fusarium* species such as *F*. *graminearum*, *F*. *cerealis*, *F*. *culmorum*, and *F*. *equiseti*. The main contamination source of ZEA are cereals, including maize, barley, oats, sorghum, and wheat, but also spices, milk, and beer [52,53,123]. The toxin is synthesized at diverse stages of food processing, like vegetation, harvesting, and storage [124]. Chemically, zearalenone is a macrolide, comprising a fourteen-membered lactone fused to 1,3-dihydroxybenzene (Figure 6).

ZEA is resistant to UV light and stable in high temperatures. A heat study has shown that toxin decomposes by 3.2% at 100 °C for 15 min and 28.5% at 150 °C for 60 min [125,126]. Two major pathways of ZEA biotransformation in animals are known. The first one is hydroxylation resulting in the formation of two stereoisomers—α-zearalenol (α-ZOL) and β-zearalenol (β-ZOL). The conversion occurs in different parts of the organism including porcine and bovine granulosa cells, swine intestinal mucosa, human intestinal Caco-2 cell line, and rat erythrocytes. The estrogenic potential of stereoisomers is different. α-ZOL is characterized by a high affinity for estrogen receptors and is more toxic than ZEA. Form β has much lower affinity for estrogen receptors and thus is nearly harmless. The next pathway is conjugation of ZEA and its metabolites with glucuronic acid. This process is catalyzed by uridine diphosphate glucuronyl transferases (UDPGT). These glucuronides are excreted into the bile and eliminated from the organism through urine and feces [55,127]. Zearalenone is a phenolic resorcylic acid lactone and its chemical structure shows a resemblance to endogenous estrogen (17β-estradiol (E2)). As a result, ZEA has estrogen-like activity and is also able to competitively bind to the related receptors [128]. As a consequence, the toxin causes estrogenic effects and induces reproductive disorders in livestock. Pigs are the more susceptible species among all domestic animals. In humans, the toxin can cause hyper estrogenic syndromes [129,130,131]. Furthermore, ZEA possesses hepatotoxic, immunotoxic, and genotoxic properties [54]. In animals, ZEA induces oocytes’ death in the follicles and a lack of ovulation. The toxin inhibits the secretion of steroid hormones, disturbs estrogenic response on the preovulatory stage, and represses the maturation of mammalian ovarian follicles [132]. In vivo studies have demonstrated that ZEA inhibits the growth of beneficial gastrointestinal microbiota. Additionally, the toxin induces an intestinal mucosal immune response, thus causing mucosal inflammation [133]. Zearalenone also induces modifications in DNA methylation and expression of genes related to nuclear receptors and metabolic pathways like IGF1, HK2, PXR, and PPARγ in the breast cancer cell line [134]. The JECFA has established a maximum tolerable daily intake (TDI) for ZEA at 0.5 μg/kg body weight [55]. According to the International Agency for Research on Cancer (IARC), zearalenone is classified in group 3 as not carcinogenic to humans [54].

## 8. Conclusions

**Figure 7:**
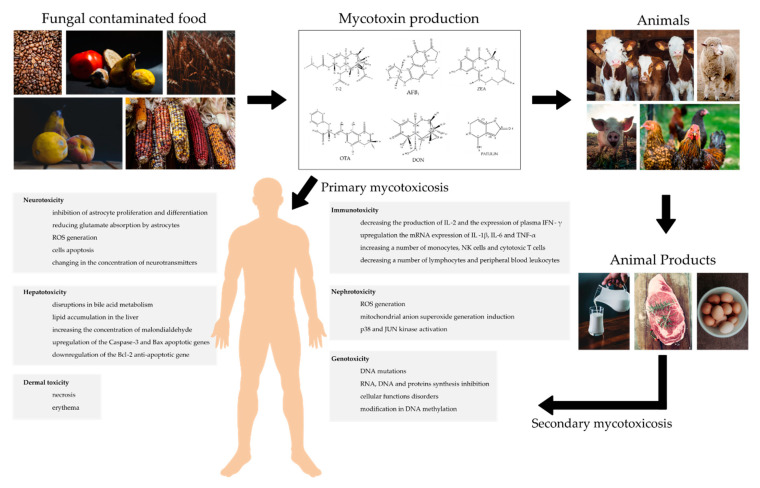
Mycotoxin mechanism of action and their impact on human organism.

Mycotoxins are poisonous, ubiquitous in chemical nature compounds, produced by various fungi species, whose occurrence in the food chain is inevitable and poses a serious problem on a global scale [135]. Human exposure to mycotoxins is common due to food and feed contamination. [136]. Mycotoxins contamination can result from poor hygienic conditions during harvest, transport, processing, or storage as well as infavorable climate. In addition to using good sanitation measures, it would be good practice to create awareness of the toxic effects of mycotoxin poisoning in humans and livestock [137,138]. Fungal contamination poses a serious threat to human and animal health, which, depending on dose and time of exposure, can lead to various ailments. The intestine is the first barrier to food contaminants and the gastrointestinal tract is the first target of mycotoxins [105]. Numerous studies focusing on the toxic actions of mycotoxins have shown that ingestion of fungal toxins may result in a variety of effects. It has been reported that mycotoxins are toxic to the nervous, immune, and reproductive systems [139]. A summary of mycotoxin mechanisms of action on the human organism is presented in Figure 7. Apart from human and animal health threat, contamination of agricultural crops with mycotoxins contributes to significant economic losses [140]. The European Commission has estimated that 5–10% of global crop losses are caused by mycotoxins, causing the loss of 2.4 billion Euro in Europe [141]. Future research should focus on generating data on epidemiological effects and long-term toxicity, especially in humans. The development of inexpensive mycotoxin detection instruments that are portable, reliable, and easy to use in the field is also an aspect to be noted. An interseting solution may be the development of new, genetically modified plants that can be resistant to fungal invasion. To maintain economic stability and agriculture, it may be beneficial to develop new protocols and strategies to compare the costs and benefits of different measures to combat fungal pathogens.

Another important issue in relation to mycotoxins is a detailed characterization of their molecular mechanism of action and its effect on animal and human health, which is necessary for the creation of strategy for prevention and therapy after poisoning.

## Figures and Tables

**Table 1:** The List of Main Mycotoxins Found in Food.

Mycotoxin	Mould Species	Food Commodity	Pathological Effects	Regulation Levels in Food	References
Aflatoxins	*Aspergillus flavus*, *A*. *parasiticus*, *A. nomius*	Wheat, walnut, maize, cotton, peanuts, maize, eggs, milk, meat	Hepatotoxicity, teratogenicity, immunotoxicity, carcinogenicity	European Union (EU): 2 μg/kg (cereals, all cereal derived products) Food and Drug Administration (FDA): 20 μg/kg (dairy animal feed) China: 5 μg/kg (barley, wheat) 20 μg/kg (corn and corn products)	[21,22,23,24,25]
Ochratoxin A	*Aspergillus ochraceus*, *A*. *carbonarius*, *Penicillium verrucosum*, *P*. *nordicum*	Coffee beans, oats, wheat, maize, wine, dried fruits, spices, eggs, meat	Nephrotoxicity, hepatotoxicity, genotoxicity, teratogenicity, immunotoxicity neurotoxicity	EU: 3 μg/kg (cereal products), 5 μg/kg (unprocessed cereal), 10 μg/kg (dried fruits), 15 μg/kg (spices) European Commission (EC): 5 ng/kg (coffee beans), 10 ng/kg (instant coffee), 0.5 μg/kg (cereal-based food) 2 μg/kg (wines) Joint FAO/WHO Expert Committee on Food Additives (JECFA): 0.1 μg/kg b. w. per week	[26,27,28,29,30,31,32,33,34,35]
T-2 toxin	*Fusarium sporotrichoides*, *F*. *poae*, *F*. *acuminatum*, *F*. *equiseti*	Barley, oats, wheat	Dermal toxicity, immunotoxicity, hepatotoxicity, neurotoxicity	EU: 100 ng/kg body weight per day	[36,37,38,39,40]
Deoxynivalenol	*Fusarium graminearum*, *F*. *culmorum*	Wheat, maize	Immunotoxicity, reproductive system toxicity, genotoxicity, gastrointestinal toxicity, neurotoxicity	JECFA: 1 μg/kg b. w. per day	[41,42,43,44,45,46]
Patulin	*Penicillium expansum*, *P*. *carneum*, *P*. *coprobium*, *Aspergillus clavatus*, *A*. *giganteus*, *Byssochlamys nivea*, *Paecilomyces saturatus*	Apples, grapes, plums, peaches, pears, tomatoes	Hepatotoxicity, nephrotoxicity, immunotoxicity, genotoxicity, teratogenicity, neurotoxicity	World Health Organization (WHO): 50 μg/kg (apples), 50 μg/L (apple juice), and 10 μg/L (young children and infants apple-based food)	[47,48,49,50,51]
Zearalenone	*Fusarium graminearum*, *F*. *cerealis*, *F*. *CulmorumF*. *equiseti*	Maize, barley, oats, sorghum and wheat	Reproductive system disorders, hepatotoxicity, immunotoxicity, genotoxicity,	JECFA: 0.5 μg/kg body weight	[52,53,54,55]

## Abbreaviations

α-ZOL	α-zearalenol
β-ZOL	β-zearalenol
AFB_1_	Aflatoxin B_1_
AFB_2_	Aflatoxin B_2_
AFG_1_	Aflatoxin G_1_
AFG_2_	Aflatoxin G_2_
AFM_1_	Aflatoxin M_1_
AFP_1_	Aflatoxin P_1_
AFQ_1_	Aflatoxin Q_1_
AST	Aspartate transaminase
ATA	Alimentary toxic aleukia
BBB	Blood-brain barrier
CYP450	Cytochrome P450
DON	Deoxynivalenol
EC	European Commission
ER	Endoplasmic reticulum
EU	European Union
FAO	Food and Agriculture Organization of the United Nations
FDA	Food and Drug Administration
FHB	*Fusarium* head blight
GLAST	Glutamate aspartate transporter
GLT1	Glutamate transporter 1
GSH	Glutathione
HBV	Hepatitis B virus
HCC	Hepatocellular carcinoma
IARC	International Agency for Research on Cancer
JECFA	Joint FAO/WHO Expert Committee on Food Additives
JNK	C-jun amino-terminal-kinase
KBD	Kashin–Beck disease
MAPK	Mitogen-activated protein kinases
MDA	Malondialdehyde
MFO	Microsomal-mixed function oxidase
MMP-9	Matrix metallopeptidase 9
OTA	Ochratoxin A
OTB	Ochratoxin B
OTC	Ochratoxin C
ROS	Reactive oxygen species
TDI	Tolerable daily intake
TJP	Tight junction proteins
UDPGT	Uridine diphosphate glucuronyl transferases
WHO	World Health Organization
ZEA	Zearalenone
